# The Role of APP *O*-Glycosylation in Alzheimer’s Disease

**DOI:** 10.3390/biom10111569

**Published:** 2020-11-18

**Authors:** Keiko Akasaka-Manya, Hiroshi Manya

**Affiliations:** Molecular Glycobiology, Research Team for Mechanism of Aging, Tokyo Metropolitan Geriatric Hospital and Institute of Gerontology, 35-2 Sakae-cho, Itabashi-ku, Tokyo 173-0015, Japan; keiko@tmig.or.jp

**Keywords:** *O*-glycan, ppGalNAc-T, APP

## Abstract

The number of people with dementia is increasing rapidly due to the increase in the aging population. Alzheimer’s disease (AD) is a type of neurodegenerative dementia caused by the accumulation of abnormal proteins. Genetic mutations, smoking, and several other factors have been reported as causes of AD, but alterations in glycans have recently been demonstrated to play a role in AD. Amyloid-β (Aβ), a cleaved fragment of APP, is the source of senile plaque, a pathological feature of AD. APP has been reported to undergo *N*- and *O*-glycosylation, and several Polypeptide *N*-acetylgalactosaminyltransferases (ppGalNAc-Ts) have been shown to have catalytic activity for the transfer of GalNAc to APP. Since *O*-glycosylation in the proximity of a cleavage site in many proteins has been reported to be involved in protein processing, *O*-glycans may affect the cleavage of APP during the Aβ production process. In this report, we describe new findings on the *O*-glycosylation of APP and Aβ production.

## 1. Introduction

Alzheimer’s disease (AD) is a progressive neurodegenerative disease in which the brain gradually atrophies, resulting in a decline in cognitive and memory function and changes in personality. The most common cause of dementia is AD. AD progresses gradually over several decades beginning from the onset of the causative changes to the appearance of clinical symptoms, making early diagnosis and treatment of this disease difficult. AD can be divided into two classes depending on how it develops. One is hereditary, known as familial Alzheimer’s disease (FAD), which is caused by mutations in the *APP*, *presenilin 1* or *presenilin 2* genes. The other is nonhereditary, known as sporadic AD, in which the disease is caused by a number of combined factors, such as lifestyle habits, including smoking, several related gene polymorphisms such as *ApoE4* gene polymorphism, the environment, and aging. More than 90% of AD is sporadic AD, the precise pathogenic mechanism of which is unknown. Here, we describe the possible involvement of glycosylation in the development of AD.

Glycosylation is a type of posttranslational modification. Glycans regulate the chemical and physical properties of proteins and consequently perform a variety of biological functions in protein quality control, molecular recognition, interaction, and protection from proteases. In other words, proper glycosylation is essential, and abnormalities in glycosylation cause various defects in organisms. In recent years, various human diseases caused by abnormalities in glycosylation have been discovered, and the number of reports of such diseases is increasing every year [1,2,3]. For example, some muscular dystrophies are caused by dysfunctions in enzymes involved in *O*-mannosyl glycan synthesis [4]. Furthermore, systemic lupus erythematosus is caused by the production of an autoreactive antibody with reduced sialylation [5], and IgA nephropathy is caused by the presence of galactose-deficient *O*-glycans on IgA1 [6]. Some diseases involving abnormal glycosylation are congenital and are caused by defects or mutations in glycan-related genes. Glycans have been shown to be involved in other diseases, although what induces these glycan changes is sometimes unclear. One of the possible causes of such changes is aging. Several reports have shown that glycans are altered with aging [7,8,9]. These glycan changes with aging may affect the onset or progression of aging-related diseases.

## 2. APP and Aβ

**Figure 1:**
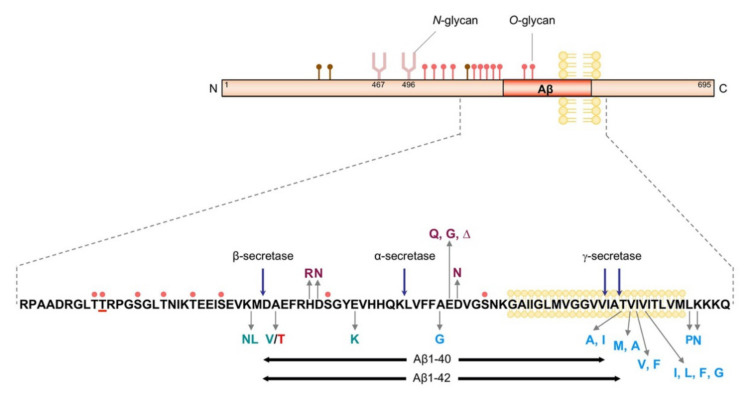
Schematic model of APP. Reported *O*-glycosylation sites and *N*-glycosylation sites are indicated. The *O*-glycan in brown means that these residues have been reported to have the potential to be modified by *O*-GlcNAc. Amino acid mutations labeled in green are mutations that affect β-cleavage. Mutations in blue affect γ-cleavage. Mutations in purple affect cohesiveness. The mutation in red is the Icelandic mutation (A673T). The pink dots indicate Ser or Thr residues that can be modified with *O*-glycans. The underlined Thr residue is *O*-glycosylated by ppGalNAc-T6.

Amyloid precursor protein (APP) is a type I membrane protein involved in AD (Figure 1) [10,11]. APP exists as three major isoforms depending on its amino acid length as a result of alternative splicing: APP770, APP751, and APP695. APP770 has a Kunitz-type protease inhibitor (KPI) domain and OX-2 domain, while APP751 contains only the KPI domain, and APP695 does not contain these domains. APP695 is predominantly expressed in neurons [10]. APP751 and APP770 are ubiquitously expressed in nonneuronal cells.

The characteristic pathological symptoms of AD are the presence of extracellular senile plaques in the cerebral cortex and the formation of neurofibrillary tangles [12]. Both are due to the abnormal aggregation and deposition of proteins, with tau accumulating in neurofibrillary tangles and amyloid-β (Aβ) accumulating in senile plaques. Aβ is a cleaved fragment of APP mainly produced from APP695 [13]. The abnormal accumulation of Aβ shows cytotoxicity and synaptotoxicity, leading to subsequent neurodegenerative processes, which finally result in neuronal death. Therefore, generation of the Aβ fragment is a key step in AD pathogenesis. APP is processed via two different pathways: the amyloidogenic and nonamyloidogenic pathways. In the amyloidogenic pathway, APP is initially cleaved by β-secretase, β-site APP-cleaving enzyme 1 (BACE1), generating membrane-associated C99, and soluble APPβ (sAPPβ) fragments. Further cutting of C99 by the γ-secretase complex, which is comprised of presenilin 1, PEN-2, APH-1, and nicastrin, within the membrane releases the Aβ fragment. Most generated Aβ fragments are Aβ1-40, but a smaller proportion are the highly cohesive and toxic Aβ1-42, which has a greater tendency to produce insoluble deposits and is a major component of the senile plaques found in the brain. In the nonamyloidogenic pathway, APP is cleaved by α-secretase and γ-secretase, secreting extracellular soluble APPα (sAPPα) fragments, preventing the generation of Aβ (Figure 1). The enzymes with α-secretase activity are ADAM9, 10, and 17, members of the a disintegrin and metalloproteinase (ADAM) family [14]. Inherited familial mutations in the vicinity of the β-cleavage site increase total Aβ production, while mutations near the γ-cleavage site increase the ratio of Aβ1-42 production to Aβ1-40 production. Newly generated, posttranslationally modified, and mature APP is transported to the cell surface [15]. After migrating to the cell surface, a portion of APP is cleaved by α-secretase and passes through the nonamyloidogenic pathway. Uncleaved APP is internalized by clathrin-mediated endocytosis from the plasma membrane. Most APP is trafficked from the endosome to the lysosome for degradation [16,17], and a portion can be recycled back to the cell surface [18] or retrograded to the trans-Golgi network (TGN). After internalization, APP is cleaved by β-secretase and γ-secretase mainly in the TGN and endosomes [19]. The Aβ produced by this pathway is secreted and accumulates extracellularly, resulting in senile plaques.

## 3. *O*-Glycans and ppGalNAc-T

*O*-GalNAc glycans are abundant glycans on mucins. Therefore, *O*-GalNAc glycans are also called mucin-type *O*-glycans. Mucins are expressed on or secreted into the mucous membranes of the gastrointestinal tract, airways, and other organ systems. Mucins contain a tandem repeat rich in Ser, Thr, and Pro; the Ser/Thr residues are highly *O*-glycosylated. *O*-Glycans on mucins contribute to tissue lubrication, protection against pathogens or chemical damage, and resistance to proteolytic enzymes. Moreover, *O*-GalNAc glycans are distributed on numerous proteins other than mucins.

**Figure 2:**
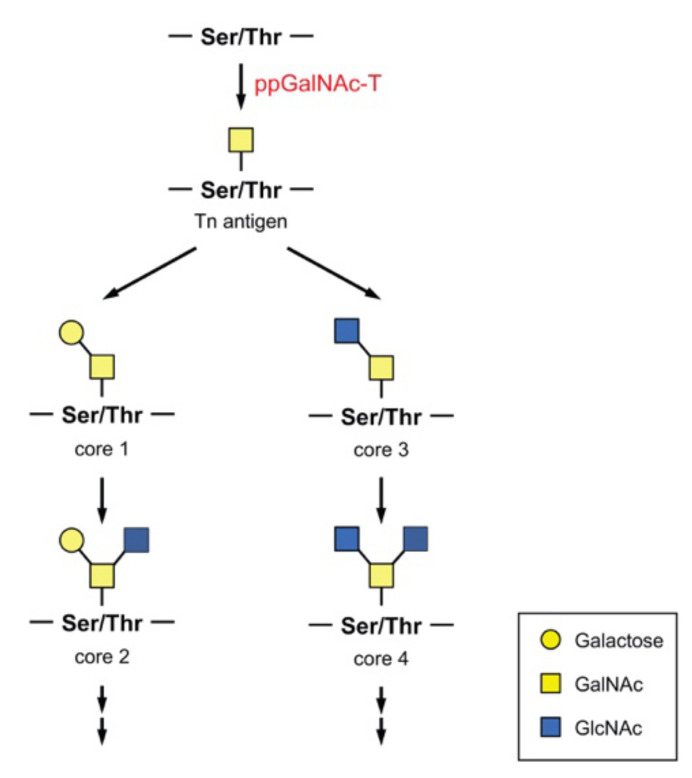
Core structures of *O*-GalNAc glycans.

A single GalNAc moiety is transferred to a Ser or Thr residue, forming a structure called the Tn antigen (Figure 2). Using this structure as a starting form, various core *O*-GalNAc glycan structures are created. The core 1 structure is formed when Gal is transferred to GalNAc through a β-1,3 bond. In contrast, the core 3 structure is formed when GlcNAc is transferred through the β-1,3 bond. When GlcNAc is added to the GalNAc moiety of the core 1 or core 3 structure through a β-1,6 bond, a core 2 or core 4 structure, respectively, is formed. These structures are extended to form a more complex structure in a highly regulated and complicated manner.

Polypeptide *N*-acetylgalactosaminyltransferase (ppGalNAc-T) is the key enzyme in *O*-glycosylation. ppGalNAc-T transfers GalNAc from UDP-GalNAc to the Ser or Thr residue of an acceptor substrate protein in the Golgi apparatus [20]. The ppGalNAc-Ts comprise a large family of approximately 20 isozymes in mammals. ppGalNAc-Ts, which are highly conserved from humans to single-celled eukaryotes but not found in yeasts or prokaryotes [21,22,23], form a large family in every species. ppGalNAc-Ts have different expression patterns in cells and tissues: ppGalNAc-T1, -T2, and -T7 are ubiquitously expressed, while other ppGalNAc-Ts have selected or restricted expression [22,24,25]. ppGalNAc-Ts are divided into groups I and II based on similarity in their amino acid sequences and substrate specificities, although ppGalNAc-T8, -T9, -T17, and -T18 have not been demonstrated to have enzymatic activity in vitro [26,27,28]. Recently, ppGalNAc-T18 was shown to regulate *O*-glycosylation through a noncatalytic mechanism [29]. ppGalNAc-T18 overexpression increased *O*-glycosylation levels to a greater extent than ppGalNAc-T2 overexpression in cells, indicating that ppGalNAc-T18 affects the expression of all *O*-glycans in cells.

Group I enzymes glycosylate both glycosylated and unglycosylated peptides and are subdivided into seven subfamilies (Ia to Ig): subfamily Ia consists of ppGalNAc-T1 and -T13; Ib consists of ppGalNAc-T2, -T14, and -T16; Ic consists of ppGalNAc-T3 and -T6; Id consists of ppGalNAc-T5; Ie consists of ppGalNAc-T8, -T9, -T18, and -T19; If consists of ppGalNAc-T11 and -T20; and Ig consists of ppGalNAc-T15. Group II enzymes prefer previously glycosylated peptides over unglycosylated peptides and are subdivided into two subfamilies (ppGalNAc-T4 and -T12 are in subfamily IIa, and ppGalNAc-T7, -T10, and -T17 are in subfamily IIb.). Substrate preference varies among isozymes [30,31,32]. Both Ser and Thr residues can be an acceptor, and a number of ppGalNAc-Ts exhibit a preference for Thr over Ser [33]. Using randomized peptide substrates, differences in Thr and Ser substrate glycosylation rates were determined. The ratio between the Thr glycosylation rate and Ser glycosylation rate varied across subfamilies, but the ratios within subfamilies were similar. This is due to differences in hydrogen bonds between Thr and bound UDP and differences in the distances between the Thr methyl group and the hydrophobic residues of the transferase active site.

ppGalNAc-Ts are type II transmembrane proteins with a short N-terminal cytoplasmic tail directed toward the cytoplasm, a transmembrane domain, a short stem region, a catalytic domain in the lumen of the Golgi, a flexible linker region, and a lectin domain localized at the C-terminus. The ppGalNAc-T family is unique in that its members contain a lectin domain that is not found in other enzymes, except human ppGalNAc-T20. Two different modes of glycopeptide recognition are likely to contribute. In one mechanism, the catalytic domain binds to Ser/Thr-*O*-GalNAc and glycosylate the neighboring site, and in the other mechanism, the lectin domain binds to Ser/Thr-*O*-GalNAc at a distant site to position the acceptor site of the glycopeptide into the catalytic domain to facilitate glycosylation [34].

ppGalNAc-Ts utilize different localization mechanisms, although all isozymes examined have been indicated to localize at the Golgi apparatus [35]. ppGalNAc-T1 and -T2 require their cytoplasmic tail and transmembrane domain. In contrast, ppGalNAc-T10 requires its transmembrane domain and luminal stem domain. ppGalNAc-T7 can localize to the Golgi by both mechanisms.

The use of a predictive algorithm showed that more than 80% of secreted proteins are expected to be *O*-glycosylated with GalNAc, and over 600 substrate proteins were identified as potential substrates of ppGalNAc-Ts by mass spectrometry [36]. Thus, *O*-glycan modifies many proteins, and the involvement of ppGalNAc-Ts in various physiological functions has become apparent. For example, ppGalNAc-T2 modifies the N-terminal ectodomain of the human δ-opioid receptor, and this glycosylation increases the half-life of the expression of functional receptor at the cell surface [37]. Site-specific *O*-glycosylation of low-density lipoprotein receptor (LDLR) and very low-density lipoprotein receptor (VLDLR) by ppGalNAc-T11 increases their affinities for low-density lipoprotein (LDL) and very low-density lipoprotein (VLDL), respectively, and enhances their uptake of ligands [38,39]. ppGalNAc-T2 is also involved in lipid metabolism. ppGalNAc-T2 has been shown to regulate high-density lipoprotein cholesterol (HDL-C) metabolism by *O*-glycosylation of phospholipid transfer protein (PLTP) and is suggested to associate with dyslipidemia and coronary artery disease (CAD) [40,41]. Congenital loss of ppGalNAc-T2 function causes low plasma HDL-C levels accompanying mental and growth retardation [42]. In Drosophila, *O*-glycosylation regulates the secretion and localization of extracellular matrix proteins involved in proper cell–cell adhesion, leading to wing formation [43]. In mice, ppGalNAc-T11 glycosylates the endocytosis receptor megalin and modulates its ligand binding in reabsorption [44]. *O*-Glycosylation by ppGalNAc-Ts has been reported to be involved in the processing of substrates, which is discussed later. Due to the involvement of ppGalNAc-Ts in various biological processes, abnormalities or deficiencies in ppGalNAc-Ts can cause various diseases, including heterotaxy [45], familial tumoral calcinosis [46,47], cancer, and many others. As mentioned above, ppGalNAc-Ts are normally localized in the Golgi, but ppGalNAc-Ts were shown to redistribute to the ER in cells following stimulation with the growth factors epidermal growth factor (EGF) and platelet-derived growth factor (PDGF) or Src microinjection [48]. Src activation upregulates COPI–dependent trafficking and ppGalNAc-Ts, but no other glycosyltransferases are selectively transported to the ER, resulting in a remarkable increase in a short *O*-GalNAc glycan, called the Tn antigen (GalNAcα1-*O*-Ser/Thr), on substrates (Figure 2). The relocation of ppGalNAc-T1 and -T2 is likely involved in cancer pathology. Cancer cell lines showed ER-localized ppGalNAc-T1 and -T2 and increased Tn antigen expression [49]. Relocation was also shown to occur in liver cancer in humans and in mice [50]. In addition, alterations in the expression of each ppGalNAc-T in different types of cancer were revealed and demonstrated to be involved in the various phases of cancer, such as tumor formation, migration, cell invasion, and metastasis [51,52,53,54,55]. Thus, incomplete glycosylation associated with changes in ppGalNAc-T expression increases the Tn antigen, which acts as a pathological marker of cancer.

## 4. *O*-Glycosylation and Protein Processing

Ectodomain shedding is a posttranslational modification in which membrane proteins, including cytokines, receptors, and adhesion molecules, are cleaved by proteases near the cell surface, releasing the extracellular domain. *O*-GalNAc glycosylation has been indicated to regulate the ectodomain shedding of several membrane proteins. For example, shedding by ADAM17 is modulated by site-specific *O*-GalNAc glycosylation [56]. Substrates of ADAM17 were examined to determine whether *O*-GalNAc glycosylation affects ectodomain shedding. Substrates with Ser or Thr residues adjacent to the cleavage site were selected, and peptides containing the cleavage site in these substrates used by ADAM17 were synthesized. Peptides were *O*-glycosylated with a variety of ppGalNAc-Ts, and glycosylation sites were analyzed by mass spectrometry. Nearly half of the peptides tested were found to be modified with one or more of the enzymes. *O*-Glycosylated peptides were subjected to a cleavage assay, which revealed that glycosylation inhibits the ADAM-mediated cleavage of several substrates, including tumor necrosis factor α (TNF-α), interleukin-6 receptor α (IL-6RA), and TGF-β receptor type-1 (TGFBR-1). In the case of TNF-α, its *O*-glycosylation at Ser80 partially inhibited cleavage by ADAM17, 9, and 10 and completely blocked processing by ADAM12. TNF-α shedding in ppGalNAc-T2-deficient cells was increased compared to that in wild-type cells. Similarly, in mice, in response to stimulation with lipopolysaccharide (LPS), ppGalNAc-T2 knockout mice showed increased TNF-α shedding compared with that of wild-type mice. Thus, TNF-α is selectively *O*-glycosylated by ppGalNAc-T2, and the *O*-glycan moiety controls cleavage by ADAM. On the other hand, a receptor tyrosine-protein kinase (ErbB4) was shown to be glycosylated by ppGalNAc-T3, and in the same study, only *O*-glycosylated ErbB4 demonstrated accelerated cleavage by ADAM10.

It has also been shown that the GalNAc-type *O*-glycosylation of Notch1 regulates ectodomain shedding [45]. The juxtamembrane peptide from Notch1 was demonstrated to be *O*-glycosylated by ppGalNAc-T11 and exhibited enhanced ADAM17-mediated ectodomain shedding. This interaction between Notch1 *O*-glycosylation and cleavage by ADAM17 is involved in the mechanism of heterotaxy.

The shedding of Cell Adhesion Molecule 1 (CADM1) is also regulated by *O*-glycosylation [57]. The ectodomain of CADM1 is released from macrophages in response to LPS stimulation. *O*-Glycosylation of Thr residues in the proximity of a cleavage site encoded by exon 8 interferes with CADM1 shedding. A variant of CADM1 including 11 amino acids encoded by exon 9 showed susceptibility to ADAM17 processing. A stretch of five nonglycosylatable residues at the C-terminal end of the 11 amino acid sequence conferred susceptibility to shedding by separating the *O*-glycan and cleavage site. A splicing variant of a member of the immunoglobulin superfamily inhibitory receptors, signal-regulatory protein α (SIRPα), was demonstrated to utilize a similar regulation mechanism in ectodomain shedding.

Proteases other than ADAM also participate in the shedding of *O*-GalNAc-modified proteins. Fibroblast growth factor 23 (FGF23), a key molecule in phosphate homeostasis, is *O*-glycosylated by ppGalNAc-T3 [47]. *O*-Glycosylated FGF23 blocked processing by furin and the secretion of intact FGF23 from FGF23-ppGalNAc-T3-coexpressing cells. Both FGF23 deficiency and ppGalNAc-T3 deficiency cause the development of familial tumoral calcinosis because reduced circulating FGF23 levels cause hyperphosphatemia. FGF23 has three *O*-glycans in the proximity of the processing site that are sequentially attached to FGF23 [46]. FGF23 containing three *O*-glycans was resistant to processing, while FGF23 with one or two *O*-glycans was not. ppGalNAc-T3 finally adds *O*-GalNAc to Thr178 in close vicinity of the processing site. This *O*-glycosylation event requires previous glycosylation at Thr171 [58].

The activation of angiopoietin-like protein 3 (ANGPTL3) is also modulated by *O*-glycosylation [59]. A Thr residue adjacent to the processing site is glycosylated by ppGalNAc-T2, which inhibits cleavage by furin.

Thus, the processing of various proteins is regulated by *O*-glycans.

## 5. Glycosylation of APP

APP undergoes posttranslational modifications, including *N*- and *O*-glycosylation [60,61,62]. APP has potential *N*-glycosylation sites at Asn467 and Asn496. An amino acid substitution study of these sites revealed that *N*-glycosylation modulates the synthesis and expression of APP [63,64]. In addition, sialylation of APP *N*-glycans enhanced the secretion of its metabolites [65]. FAD mutations (the Swedish type and London type) caused alterations in *N*-glycan structure, although the mutation sites were located apart from the *N*-glycosylation sites [66]. Both mutated APPs contained much higher amounts of bisecting GlcNAc and core-fucose than wild-type APP. The mRNA expression of β-1,4-mannosyl-glycoprotein 4-β-*N*-acetylglucosaminyltransferase (GnT-III), a glycosyltransferase responsible for bisecting GlcNAc synthesis, in the AD brain was higher than that in the control brain [67]. Experiments using GnT-III-overexpressing cells and knockout mice revealed that bisecting GlcNAc affects Aβ generation [67,68].

It is rather easy to identify *N*-glycosylation sites because *N*-glycosylation takes place on Asn at a consensus sequence Asn-X-Ser/Thr (X is any amino acid other than Pro). *O*-glycans are known to be attached to the hydroxyl groups of Ser or Thr residues; the *O*-glycosylation consensus sequence has not been elucidated, and the *O*-glycan-binding site cannot be identified from amino acid sequences. Several studies have examined the *O*-glycosylation sites of APP utilizing mass spectrometry. Human APP695 was expressed in CHO cells, secreted APP was purified from the culture medium; *O*-glycosylation sites and glycan structures were identified from this purified APP [69]. Thr291, Thr292, and Thr576 of APP695 were modified with sialylated core 1 type *O*-glycans. Some reports have examined *O*-glycosylation by recovering sAPP or Aβ from human cerebrospinal fluid [70,71]. Consequently, a number of additional *O*-glycosylation sites have also been reported: Thr633, Thr651, Thr652, Ser656, Thr659, Thr663, and Ser667 in APP770 correspond to Thr558, Thr576, Thr577, Ser581, Thr584, Thr588, and Ser592, respectively, in APP695. Tyr681 was also found to be *O*-glycosylated in human and cat short Aβ peptides (Aβ1-15 to Aβ1-20) [70,71]. Only APP770 has an *O*-glycan at Thr353 within the OX2 domain, in addition to the sites listed above [72]. The *O*-glycosylation of APP occurs after *N*-glycosylation and is necessary for the proper transport of APP [61]. A certain amino acid substitution resulted in impaired *O*-glycosylation and the intracellular accumulation of APP. As a result, Aβ production was decreased, indicating that APP processing occurs following *O*-glycosylation.

**Figure 3:**
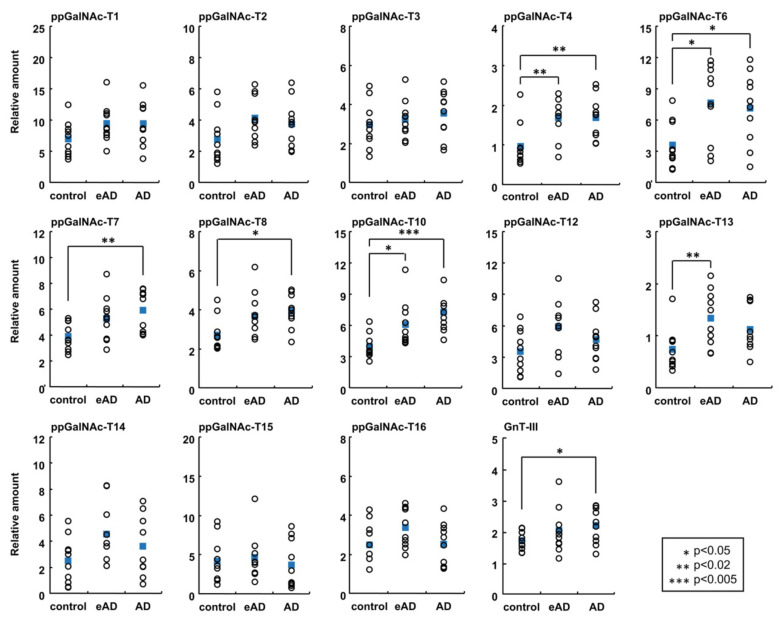
The expression of glycosyltransferases in AD brains by real-time PCR [67,75]. The results for isozymes in the ppGalNAc-T family and GnT-III are shown. Open circles indicate individual samples, and blue squares indicate average values for each group. eAD: early-stage AD.

Notably, ppGalNAc-T1, -T2, -T3, and -T13 exhibit enzyme activity for the transfer of GalNAc to APP, but differences in the preference of ppGalNAc-T isoforms for glycosylation sites on APP have been found [73]. Furthermore, ppGalNAc-T2 and -T4 were demonstrated to modify APP by *O*-GalNAc modification [74]. The expression of ppGalNAc-Ts in the brains of patients with Alzheimer’s disease was examined by real-time PCR (Figure 3) [75]. Among the 13 ppGalNAc-Ts detected, the mRNA expression levels of ppGalNAc-T4, -T6, and -T10 were significantly increased in association with the progression of AD, and all these isozymes showed enzymatic activity against APP. The level of ppGalNAc-T3 expression was not changed in the AD brain, although ppGalNAc-T3 is highly homologous to ppGalNAc-T6.

Furthermore, APP is modified by another type of *O*-glycosylation, *O*-GlcNAcylation. The monosaccharide *O*-GlcNAc is added to Ser or Thr by *O*-GlcNAc transferase (OGT) and reversibly removed by *O*-GlcNAcase (OGA). In contrast to other types of glycosylation, *O*-GlcNAcylation frequently occurs within the nucleus and cytoplasm [76]. *O*-GlcNAcylation and phosphorylation often compete because they target the same residues [77]. APP is modified by *O*-GlcNAcylation [78]. The *O*-GlcNAcylation on APP promotes localization to the cell membrane and nonamyloidogenic processing [79,80]. Thr567, the most promising candidate for *O*-GlcNAc modification, regulates APP trafficking and processing [81]. However, the addition of *O*-GlcNAc by OGT occurs at the intracellular proteins or intracellular domains of membrane proteins, since the OGT and OGA enzymes function in the cytoplasm. It has been reported that EGF-domain *O*-GlcNAc transferase (EOGT) is responsible for *O*-GlcNAc addition to the extracellular domain of Notch1 [82]; EOGT may also be responsible for the *O*-GlcNAc modification of APP.

## 6. *O*-Glycosylation and Aβ Production

The modification of APP by ppGalNAc-Ts has been shown to be involved in APP processing. For example, a close relationship between ppGalNAc-T2 and Aβ production was reported [73]. Treatment with luteolin, a citrus flavonoid, inhibited ppGalNAc-T2 activity and reduced Aβ production in the cells coexpressing APP and ppGalNAc-T2. Luteolin was demonstrated to be a competitive inhibitor of the active form of ppGalNAc-T2 by X-ray structural analysis and ^1^H NMR experiments. As mentioned above, other ppGalNAc-Ts, such as ppGalNAc-T1, -T3, and -T13, have enzyme activity for the transfer of GalNAc to APP [73]. In addition to its inhibition of ppGalNAc-T2, luteolin inhibited ppGalNAc-T3 and decreased *O*-glycosylated APP but had no effect on ppGalNAc-T1 or -T13. Luteolin treatment was shown to reduce Aβ production in the brains of APP/PS1 transgenic mice [73]. Another report demonstrated that the treatment of Tg2576 mice (mice overexpressing the AD-related ‘Swedish’ mutant APP) with luteolin resulted in a significant reduction in soluble Aβ and cerebral amyloidosis. However, luteolin was suggested to inhibit GSK-3 activation, leading to an increase in presenilin 1 phosphorylation in this case [83].

In our previous study, the coexpression of APP and ppGalNAc-T6 markedly reduced Aβ generation [75]. This is because β-cleavage was substantially decreased while the expression level of membrane-bound APP remained unchanged. A ppGalNAc-T assay using a synthetic peptide containing sequences around the β-cleavage site revealed that ppGalNAc-T6 preferentially transfers GalNAc to the Thr577 residue of APP695. The neighboring residue Thr576 was not modified by ppGalNAc-T1, -T4 or -T6, although both Thr576 and Thr577 were reported to be *O*-glycosylated [70,71]. There are some possible explanations for the decrease in β-cleavage. One is that *O*-glycosylated APP is not transported properly to the appropriate location for β-cleavage. Another possibility is that BACE1 is unable to approach *O*-glycosylated APP by conformational change. The upregulation of ppGalNAc-T6 observed in the AD brain and the protective effect of ppGalNAc-T6 against Aβ generation seem to be inconsistent. Further study is needed to unravel this contradiction.

*O*-GlcNAcylation has also been associated with APP processing [81,84]. *O*-GlcNAcylation at Thr576 regulates the trafficking of APP to the cell surface and decreases the endocytosis rate. As a result, *O*-GlcNAcylated APP is cleaved by α-secretase and produces nonamyloidogenic Aβ.

Whole-genome sequencing of Icelanders revealed a novel coding mutation in the *APP* gene (corresponding to Ala598 of APP695) termed the Icelandic mutation (A673T) [85]. This mutation was also found in Norwegian, Finnish, and Swedish populations but is extremely rare in other populations [86,87]. Interestingly, this mutation was found more frequently in an elderly control group than in an AD group. Moreover, cells transfected with APP harboring the A673T mutation produced ~50% less sAPPβ and ~40% less Aβ than wild-type APP-transfected cells [85]. The A673T mutation was reported to reduce APP β-secretase cleavage and consequently Aβ production [88]. It has also been reported that Aβ carrying the A673T mutation is less cohesive than wild-type Aβ [89]. Further study elucidated that these reductions in APP cleavage products were due to elevated BACE1 cleavage at the β’-site [90]. From these results, the A673T mutation appears to have a protective effect against AD. Since position 673 is located in close vicinity of the β-site, it is conceivable that this amino acid substitution influences cleavage by BACE1. Indeed, the APP and BACE1 interaction in neuronal cells was attenuated by the A673T mutation [91]. One possible explanation for this finding is that this gene mutation caused the *O*-glycosylation of Thr673 and that the addition of an extra glycan led to changes in APP localization, although there are other possibilities.

## 7. Conclusions

Over the last 15 years or so, since the first report on *O*-GalNAc glycan modification and FGF23 processing was published, the shedding of several other substrates has been demonstrated to be regulated by *O*-GalNAc glycans. In this review, we have focused on the role of *O*-GalNAc glycans in protein processing and described the effect of glycans on the shedding of APP. The modification of *O*-glycans by ppGalNAc-T6 and -T2 seems to be involved in Aβ generation. In our experiments, ppGalNAc-T6 was found to be involved in Aβ production, and *ppGalNAc-T6* mRNA expression was increased in the brains of AD patients, although whether this is the cause or result of the disease has not been elucidated. Furthermore, ppGalNAc-T6 appears to act to both inhibit and promote cancer [92,93]. ppGalNAc-T2, another *O*-type glycosyltransferase involved in APP processing, also appears to act in both modes with respect to cancer [94,95]. For these reasons, it will be difficult to increase or decrease a single ppGalNAc-T to treat AD. Maintaining the overall glycosylation status at a state similar to that in the young may help reduce the development of aging-related diseases such as Alzheimer’s disease. However, further research is needed.
